# Fracture of Human Femur Tissue Monitored by Acoustic Emission Sensors

**DOI:** 10.3390/s150305803

**Published:** 2015-03-10

**Authors:** Dimitrios. G. Aggelis, Maria Strantza, Olivia Louis, Frans Boulpaep, Demosthenes Polyzos, Danny van Hemelrijck

**Affiliations:** 1 Department of Mechanics of Materials and Constructions, Vrije Universiteit Brussel, Pleinlaan 2, 1050 Brussels, Belgium; E-Mails: maria.strantza@vub.ac.be (M.S.); frans.boulpaep@vub.ac.be (F.B.); danny.van.hemelrijck@vub.ac.be (D.H.); 2 Department of Radiology, UZ Brussel, Vrije Universiteit Brussel, Laarbeeklaan 101, 1090 Brussels, Belgium; E-Mail: olivia.louis@uzbrussel.be; 3 Department of Mechanical Engineering and Aeronautics, University of Patras, Panepistimioupolis Rion, 26500 Patra, Greece; E-Mail: polyzos@mech.upatras.gr

**Keywords:** mechanical loading, bone, fracture pattern, torsion, bending, RA, frequency, monitoring

## Abstract

The study describes the acoustic emission (AE) activity during human femur tissue fracture. The specimens were fractured in a bending-torsion loading pattern with concurrent monitoring by two AE sensors. The number of recorded signals correlates well with the applied load providing the onset of micro-fracture at approximately one sixth of the maximum load. Furthermore, waveform frequency content and rise time are related to the different modes of fracture (bending of femur neck or torsion of diaphysis). The importance of the study lies mainly in two disciplines. One is that, although femurs are typically subjects of surgical repair in humans, detailed monitoring of the fracture with AE will enrich the understanding of the process in ways that cannot be achieved using only the mechanical data. Additionally, from the point of view of monitoring techniques, applying sensors used for engineering materials and interpreting the obtained data pose additional difficulties due to the uniqueness of the bone structure.

## 1. Introduction

Acoustic emission (AE) is a non-invasive technique used in several situations to monitor the fracture behaviour of different types of materials. Piezoelectric sensors are attached on the surface of the material being tested in order to record the transient elastic waves generated by cracking events. This provides valuable input on the failure development from the start of loading and certainly before the fracture is visible [1]. The values of AE parameters and the accumulated number of recorded activities are correlated to the sustained load and the damage condition of the materials [2,3,4,5]. Furthermore, indices based on the energy or amplitude of the waveforms help to characterize the intensity of fracture and hopefully make projections for the future life of engineering components [6,7]. In different types of materials, AE has shown a capacity to characterize the fracture mode corresponding to different stresses (normal *vs.* shear) or between different mechanisms like cracking and fibre pull-out or delamination of successive layers. There are plenty of examples in materials like concrete, metals, ceramics, composites and rock [2,3,4,5,6,7,8]. However, the application of AE in human bone tissue entails specific difficulties. A serious one is the limited number of tests that can be conducted since the samples are excised from cadavers. Literature surveys regarding the use of AE in the biomedical field can be found in Browne *et al.* [9] and Shrivastava and Prakash [10]. Specific studies have used AE for characterization of bone behaviour. Ossi *et al.* [11] reported that transmission of the AE energy in bovine bones depends on the amount of saturation. In addition, Agcaoglu and Akkus [12] claimed that during fatigue loading, AE indicates the onset of failure in the human tibia cortical bone. Another study from Van Toen *et al.* confirmed the capability of AE signals to detect time of injury and to discriminate between failures of different spinal components in dynamic loading. The AE signals from compressive bone fractures were linked with higher amplitudes and frequencies than those from tensile failures of ligaments [13]. Substantial AE was recorded during tensile loading of the anterior cruciate ligament (ACL, knee joint), showing that along with the elongation of the tissue, the frequency content of AE was also increased [14]. In a pioneering work in the 1980s, AE was monitored on a simplified prosthetic system implanted into cadaveric tibia and femur bones [15]. It was found that the AE activity (and therefore, “the propensity for failure”) increases at a lower strain rate and that higher amplitude emissions were recorded for higher load. Furthermore, AE has been applied for assessment of knee joint osteoarthritis and friction [16] and monitoring of hip implants [17,18,19].

Apart from the relatively limited number of experimental works, another difficulty is the geometry of the specimens, which usually includes curvatures and poses problems in terms of positioning and stability of the AE sensors. An important factor is also the interpretation of the results. Since the background is not strong in the field (compared to the exhaustive studies in concrete, metal or other engineering materials), it is not easy to explain the trends or the values of AE parameters. Despite the difficulties, these studies are very significant, since bone fractures and specifically fractures of the femur (hip fracture) are a very common cause of eventual loss of life, or at least loss of quality of life, for millions of aged people [20]). In any case, the understanding of the pattern according to which the tissue is broken as well as the mechanical properties of the tissue are important for medical doctors who are studying their surgical repair. Fracture in bone tissue is a very complicated process that depends on several parameters: the relative fraction of bone tissue over void space, the geometrical arrangement of the bone tissue, *i.e.*, “architecture”, the thickness as well as the mechanical properties of the tissue, and the applied load configuration [21,22]. While the static stress and strain fields of long bone under various types of loading (axial, torsional, bending) can be simulated reliably [23], the simulation of damage propagation becomes much more complicated due to the abovementioned microstructure-related reasons. Therefore, AE monitoring can shed light on the process from the moment of first cracking to the ultimate failure. It should be stressed, however, that apart from AE, considerable effort has been made in the ultrasonic assessment of bone condition, which helps in the diagnosis of osteoporosis or healing of bones [24,25,26,27]. Vibrational behaviour examined by PZT patch sensors has also been utilized in long femur bones in order to differentiate between intact bone and bone with different sizes of cracks [28].

In addition to their significance, AE tests in bones involve a great scientific challenge related to experimental techniques. The mechanical parts of the test, as well as the monitoring, pose certain difficulties, and the way to overcome these is not straightforward. From the AE point of view, the challenge is mainly the details of sensor placement and, certainly, the interpretation of the activity observed due to (so far) limited experience.

In this study, results of fracture tests on whole human femur bones with AE monitoring are described. The setup applies a mixed bending-torsion monotonic loading up to failure. The AE activity shows the onset of micro-cracking as well as its development. AE parameters like the frequency content and rise time exhibit certain shifts with the increase of load, showing that the fracture mechanisms are not stable throughout loading. Additionally, specimens that obviously fractured with different patterns demonstrate major changes in their AE activity. This is in accordance with previous studies, where it was shown that different fracture orientations relatively to the osteons resulted in different fracture toughness [29], implying that monitoring of the released energy could record these changes during fracture. Discussion also extends to the correlation between AE parameters and the thickness of the cortical shell.

## 2. Experimental Details

This study was performed on 11 femur specimens excised from cadavers. The specimens were supplied by the Anatomy Department of the School of Medicine of the Vrije Universiteit Brussel (Brussels, Belgium) and had been preserved using a formol solution injection into the vessels.

**Figure 1:**
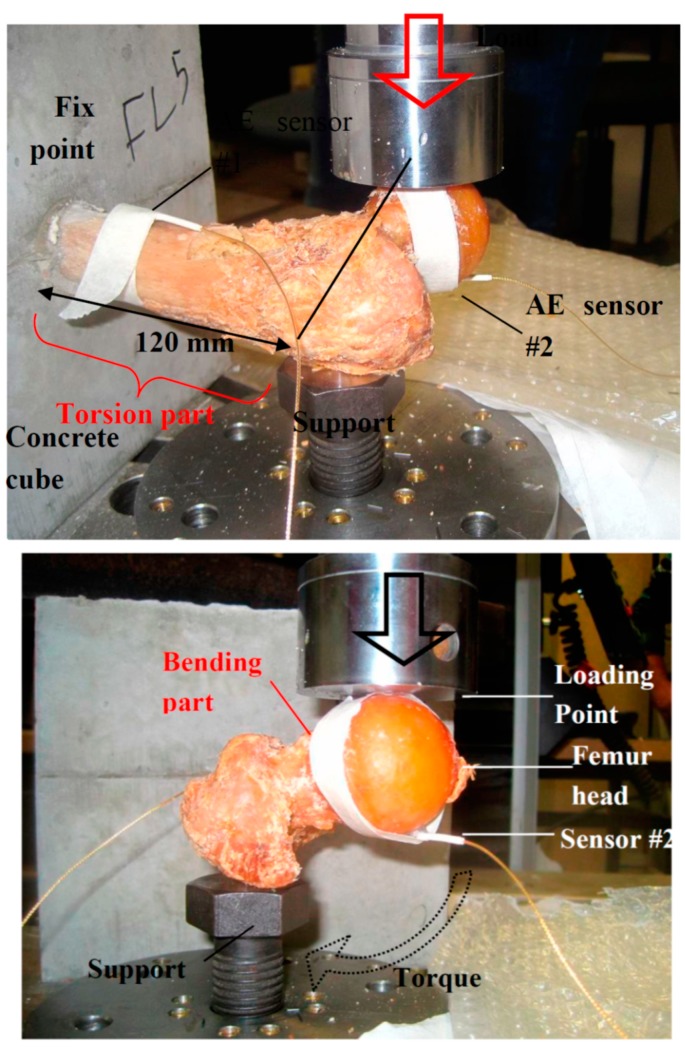
Different angle views of the test.

In order to perform the test, a large part of each bone was cast in concrete, as seen in Figure 1. The “head” of the femur was 120 mm outside from the fix point in all specimens. To avoid fracture at the fix point as a cantilever under bending, a support was provided in the main body of all specimens (point of minimum elevation) by a metal bolt (Figure 1 top and bottom). The monotonic load was applied by a piston, resulting in a vertical force on the head. The geometry resulted in a combination of bending and torsion, also leading to different fracture patterns as will be discussed in a next section. The clinical relevance of the selected loading geometry is that the experimental design, resulting in a combination of bending and torsion, was chosen in order to mimic neck fractures occurring as a consequence of falls.

Concerning the AE monitoring, two broadband transducers were used. The first was placed near the fix point and the second underneath the head (Figure 1). The exact position of the sensors could not be identical in all specimens due to differences in geometry and local curvature. The sensors were “pico” sensors with a relatively broadband response and peak sensitivity at 450 kHz. The specific sensors were selected due to their response but also due to their small size (diameter of 5 mm) which enabled placement on the curved surfaces of the specimens. The AE signals were pre-amplified by 40 dB and acquired in a Mistras micro-II board (totally 8 channels) with sampling rate of 10 MHz. The threshold was set at 30 dB, while the peak and hit definition times (PDT and HDT) were 200 μs and 800 μs respectively. Acoustic coupling was improved by the use of Vaseline grease between the sensors and the contact points on the femur, while tape was used to secure the sensors during the experiment. Since two sensors were used, the capability of linear localization was assessed despite the complicated and anisotropic geometry. It was seen that excitations produced by pencil lead breaks before loading in three areas (femur head, middle of diaphysis and fix point) were correctly classified in the actual regimes (e.g., close to sensor 2, in the center between the sensors, and close to sensor 1 respectively). More accurate localization would require multiple sensors as in the work of Qi *et al.* [19]. Although wave propagation is very complicated and different modes are created as measured in a recent ultrasonic study on the same specimens [30], a representative pulse velocity resulting in acceptable localization was 3500 m/s.

**Figure 2:**
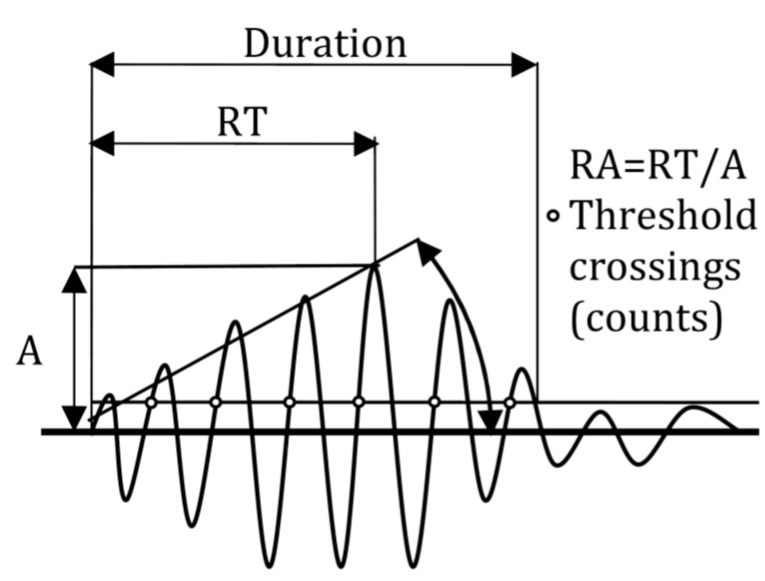
Typical AE waveform.

A typical AE waveform is seen in Figure 2. Amplitude (A) is generally an important parameter as it correlates to the intensity of the fracture incident. Energy stands for the area under the rectified waveform. Additionally, the rise time (RT) is the delay between the first threshold crossing (or first count) and the peak amplitude. Rise time over amplitude (RA value) is also important, defined as RT divided by A and is measured by μs/V. Average frequency (AF) is the number of threshold crossings over the duration and it is a good approximation of the frequency content of the waveform. Other important frequency parameters like the “central frequency” and the “peak frequency” require the FFT of the waveform in order to calculate the centroid and the frequency with the maximum magnitude respectively.

## 3. Results and Discussion

### 3.1. AE Activity

**Figure 3:**
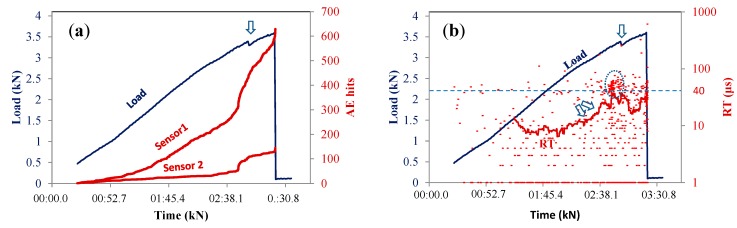
Load history and (**a**) cumulative AE activity of different sensors; and (**b**) rise time (RT) of sensor #1, for femur specimen #8. The RT solid line in (**b**) is the sliding average with window of 30 points.

**Figure 4:**
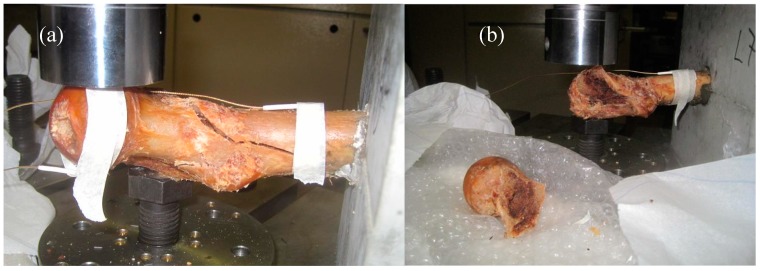
Fractured femur specimens: (**a**) diagonal crack through the diaphysis (#8) and (**b**) femur head detachment (#7).

The total AE activity of indicative femur specimens is discussed herein. The cumulative number of hits recorded from the two sensors separately is depicted with the load history in Figure 3a. For the early stage of loading, there is no activity showing that there was no contribution of random noise. The activity started at the load level of 0.5 kN which is 14% of the maximum load and is the manifestation of initiation of micro-cracking phenomena. The recording rate of AE hits over time was continuously increasing until a point when the sensors registered a sharp increase. A few seconds later, a macro-fracture event was evident by a transient load drop (from 3.38 to 3.29 kN, see the arrow). After this drop, the specimen did not bear much higher load, being macroscopically broken at 3.6 kN. These macroscopic fracturing events were seen in all specimens, resulting in a strong load drop and vertical increase of the recorded AE activity. Apart from the information on the onset of cracking, important trends can be seen through AE waveform parameters. For example, in Figure 3b, the RT values of all hits of sensor 1 (close to the fixing point) are depicted for the same specimen. The majority of them are up to 20 μs, with only a few being up to 60 μs. At around 2 min in the loading, the sliding average line obtains a steady increasing trend (see twin arrows in Figure 3b) and just before the first strong fracture event, a group of points between 40 and 60 μs are recorded (dashed ellipse), causing an even stronger increase on the sliding average line. Another fluctuation is noted at the moment of load drop. In the engineering field, an increase of RT usually signifies shift from tensile cracking to shearing [1,8,31,32,33]. Shearing may be expressed by fiber pull-out, delamination or cracking due to shear stresses. Although the experience in AE testing in human bones is limited, the type of test including both bending and torsion moments does not exclude different dominant mechanisms for individual specimens. Indeed, the fracture pattern of this specimen is shown in Figure 4a. The crack ran diagonally through the diaphysis with no apparent connection to the femur head, which establishes a reasonable connection to torsion of the diaphysis.

**Figure 5:**
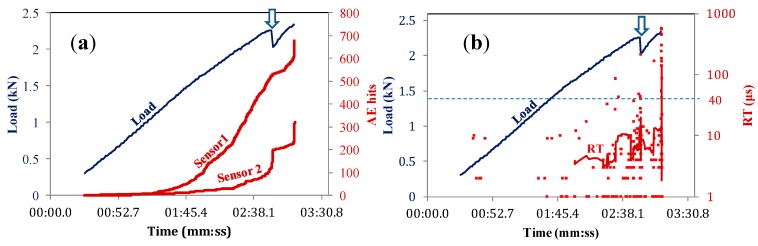
Load history and (**a**) cumulative AE activity of different sensors and (**b**) RT of sensor #2, for femur specimen #7. The rise time solid line in (**b**) is the sliding average with window of 30 points.

Another example of cumulative AE activity is seen in Figure 5a concerning another sample. The continuous activity started at 13% of the ultimate load similar to the previous specimen. The AE rate increased for sensor #1 earlier than #2, indicating that more cracking activity was occurring near the fix point. However, the activity of sensor #2 placed beneath the head started to increase at approximately 2:20, which was the precursor of the macroscopic fracture event at 2:52 s (again indicated by an arrow). This vertical increase of the AE activity of sensor #2 is in agreement with the visual observation of the specimen after the test, which fractured near the head, as seen in Figure 4b. Taking a look at the RT in Figure 5b, it is obvious that the level of values does not exhibit steady strong trends as in the previous case, rather than momentary fluctuations being at lower levels in general. Specifically, only 6.2% of the hit populations are above 40 μs (see reference dashed horizontal line), while in the previous case which fractured by a diagonal crack, the corresponding percentage was much larger at 21.1%.

The load at which AE started to be recorded in all specimens is seen in Table 1. This information is related to the moment when systematic cracking was initiated within the specimens. Despite the inherent heterogeneity and the differences between samples, most of them exhibit the start of micro-cracking in between 10%–15% of the maximum load, with only a few exceptions.

**Table 1:** Load at the onset of AE recording as a percentage of the maximum sustained load.

Specimen #	Load (%)
1	6.7
2	15.4
3	9.2
4	29.6
5	10
6	13.9
7	13.1
8	14.1
9	38.3
10	16.7
11	13.6

### 3.2. Correlation with Thickness

**Figure 6:**
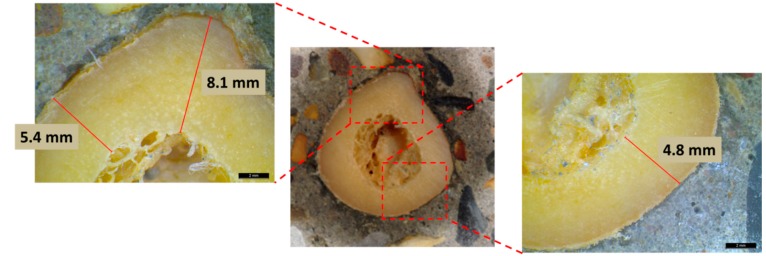
Cross section of a femur specimen with different thickness measurements.

The thickness of the femur consists of three layers with high heterogeneity. Cross-sections of cortical bone clearly show age-dependent differences [34,35]. In this study, the age of the cadavers was between 73 to 95 years old. Therefore, the cortical bone area is expected to be relatively low since the compact bone cross section decreases with age. Five specimens were cut vertically to study their cross section by microscope, and an example is given in Figure 6. The thickness measurements are based on the cortical layer, which seemingly is the outer thick part of the sections. The value of thickness was measured as average of several measurements in the radial direction. It is clear from the photographs of the microscope that the thickness in the femur is not uniform due to the inhomogeneous geometry of the tissue. For the specific case, different measurements range between 4.8 and 8.1 mm with an average value of 5.9 mm.

**Figure 7:**
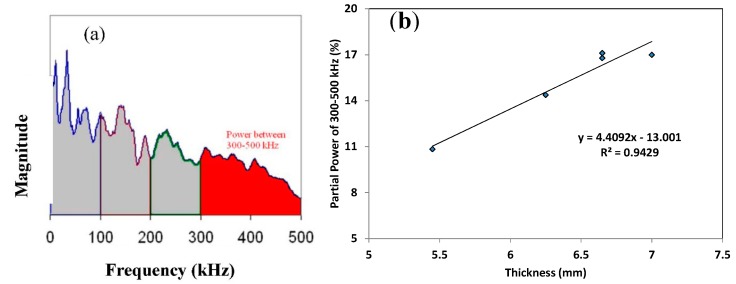
(**a**) Correlation between partial power of the band 300–500 kHz with the average thickness of the bones; (**b**) Illustration of partial power feature.

In search of parameters that are sensitive to the material’s condition, the energy content of different frequency bands was analyzed. These bands were defined as shown in Figure 7a from previous experience up to 500 kHz. Content in higher bands is not expected due to attenuation of the material. Concerning the five samples that were studied in the microscope, the strongest correlation is noted between the energy of the band between 300–500 kHz and the average bone thickness and it is presented in Figure 7b. This partial power is the percentage of the area of the specific frequency band divided by the total band of 0–500 kHz, shown in Figure 7a. Specimens with thicker layer of cortical bone (around 7 mm in average) exhibited AE with 17% of its energy in the highest frequency range, between 300 and 500 kHz. As the average thickness decreased to 5.5 mm, the energy of this high frequency band decreased to almost 10%. This correlation is certainly preliminary and should be validated in larger number of specimens. However, it is encouraging in the sense that parameters obtained by a monitoring technique like AE, exhibit relation to a physical property of the tissue. The fact that the sensors’ peak sensitivity is within this range (*i.e.*, 450 kHz) could be related to the strength of the correlation but cannot be taken for granted as the attenuation of the material is also dominant on the final frequency content of the received signal.

### 3.3. Fracture Mode Influence on AE Parameters

**Figure 8:**
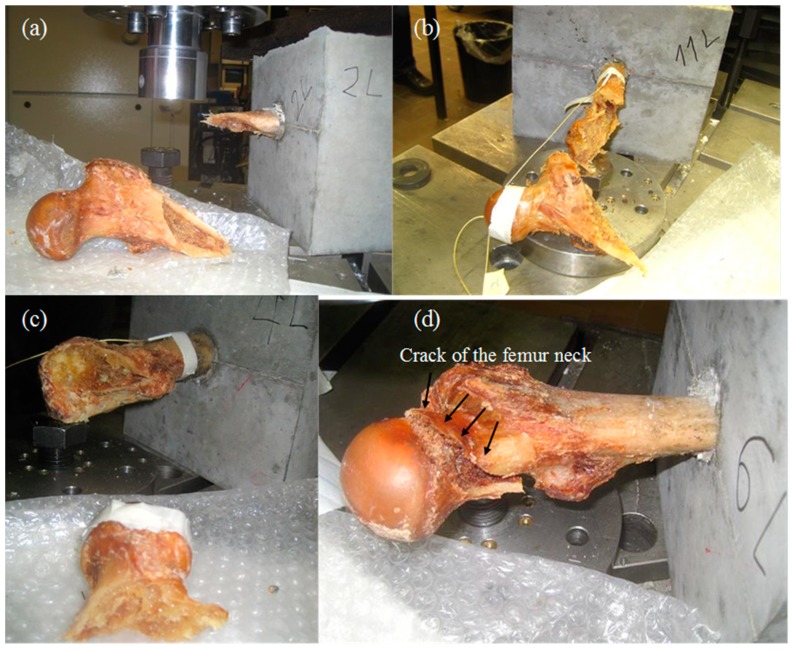
Different fracture patterns: (**a**) and (**b**) through the femur body; (**c**) and (**d**) on the femur head.

As mentioned earlier, the fracture test includes three points of boundary conditions. These are the fix point on the concrete cube, the support provided by the bolt and the load application point. Due to the distance between the load point and the supporting bolt the load results in torsion for the main bone (diaphysis), as seen in Figure 1. However, the same force applies a bending moment on the part of the femur between the loading point and the supporting bolt (mainly the spherical head and its connection to the rest of the body, which is called the “neck”). Therefore, the loading can be regarded as a mixture of bending and torsion. Reasonably, it can be argued that if the fracture occurs between the supporting bolt and the fix point, it includes strong torsion character triggered by the shear stresses on the load bearing cortical bone. On the other hand, if the fracture occurs at the level of the neck, this is closer to bending failure where normal stresses play more important role. Below, some examples of the different fracture patterns are depicted. Figure 8a,b concerns fracture through the femur diaphysis. In the case of Figure 8a, the specimen was totally separated into two parts by a diagonal crack which nearly reached the fix point. The same pattern is seen in Figure 8b, with the specimen nearly separated in two, while the area around the head has not sustained obvious damage. On the other hand, Figure 8c,d shows two examples of femur neck fracture. In the case of (c) the head was totally separated from the rest of the specimen, while in (d) the crack formed around the head and propagated slightly into the body of the bone. Based on the geometry of the test and the final fracture pattern, it can be argued that the first two cases of fracture were mostly due to torsion/shear stresses, while the two latter were mostly due to normal stresses of bending of the femur neck.

As mentioned, one of the benefits of AE for most engineering materials is that it helps to characterize the fracture mode. It has been established that fracture due to shear stresses emits AE waveforms of long duration, high RA values and low frequency content. This has been repeatedly examined in engineering materials like concrete [8,31,32,33]. Therefore, since two distinct fracture patterns were also observed in these tests, it was deemed appropriate to examine if the above connection between AE waveforms and fracture mode holds for human bone tissue as well. For the purpose of the analysis, the signals during the major fracturing moments of the specimens were used. This is because it is certain that micro-fracturing occurred in different places of the specimen throughout the whole duration of loading. By including the total population, trends would be mixed and conclusions would be difficult. However, focusing on the moment of load drop, it is certain that the recorded AE corresponds to the fracture mode responsible for the ultimate failure. This way, comparisons can be made between specimens that were fractured following different patterns. The data were plotted in the AF-RA axes as typically done for other engineering materials [31,32,33]. Each of the following figures include populations of two specimens (one with torsion crack and one with bending crack), in order to make the comparisons easier. Later, the average values of some indicative AE parameters are depicted in Table 1.

**Figure 9:**
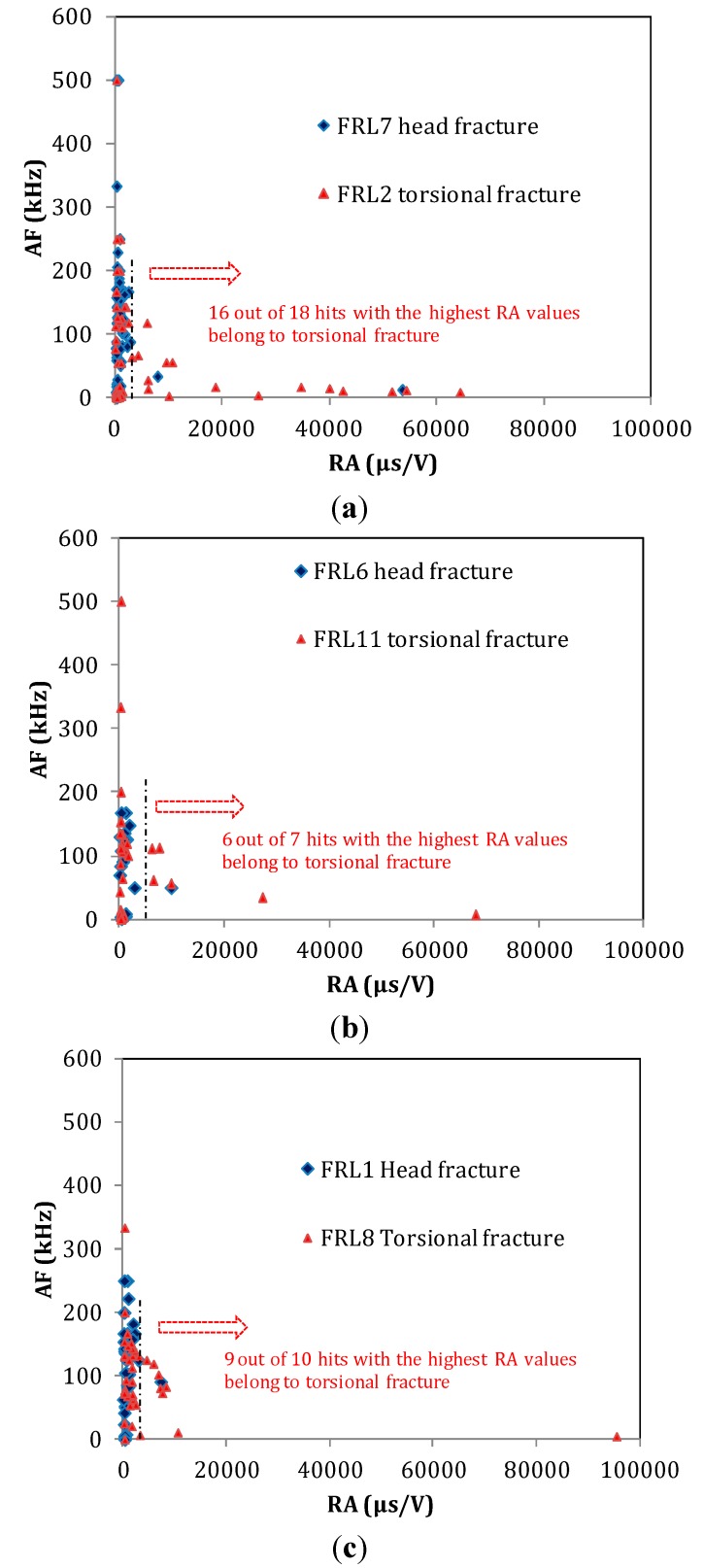
Plot AF *vs.* RA for three pairs of femur specimens, as monitored by sensor 2 (at the head). Couples for comparison: (**a**) specimen 2 and 7; (**b**) specimens 6 and 11; (**c**) specimens 1 and 8.

In all cases in Figure 9, most of the points are concentrated at a narrow zone of low RA values spanning frequencies mostly up to 300 kHz. Only a small part of the population extends to higher RA values. For all the comparisons in Figure 9a–c, it is obvious that the points occupying the lower-right part of the plot (high RA and low AF) belong to the torsion fracture rather than bending. Characteristically, in Figure 9a, 89% of the AE hits with the highest RA value (16 out of 18) belong to torsion fracture. For the next subfigures, the trend remains (6 out 7 hits and 9 out of 10 for the cases in Figure 9b,c respectively). In total, 85% to 90% of the activity with the highest RA is due to fracture of the main body, while only 10%–15% is due to the femur neck bending fracture. Therefore, it is reasonable to conclude that shear stresses (in this case due to torsion loading of the diaphysis) lead to fracturing events with longer RT and higher RA compared to bending of the femur neck, which results in strong normal stresses (tensile at the top and compressive at the bottom of the neck). It is mentioned that in an earlier study of bending-torsion load in human femur samples including unloading and reloading, RT has been identified as a parameter to distinguish between cracking and friction of the existing crack banks [17]. It was concluded that shorter signals belong to cracking and longer to friction. However, exact values are not supplied, so direct comparisons are not possible even if differences in terms of AE systems and loading conditions could be disregarded.

Table 1 shows the average values for some indicative AE parameters for the six specimens which exhibited a quite clear pattern of fracture either due to bending of the neck or torsion of the diaphysis. As aforementioned, the values are not the average of the whole population, but of the population during the moments of serious fracture, (accompanied by load drops) where the activity line increases almost vertically. The activity of the two sensors is separated in order to enable comparisons between the same sensors, which were placed at similar locations in the specimens. The differences are clearer for RT and RA, while for AF there is overlap. Specifically, bending failure monitored by any sensor resulted in RT lower than 30 μs, while torsion failure between 30 and 85 μs. Correspondingly average RA values for bending are typically below 2000 μs/V, while for torsion fracture the values are higher than 2000 μs/V with the exception of specimen 8 for channel 1. For frequency, differences are not as strong, but still there is a shift of more than 20 kHz in average between the two modes of fracture (higher for bending). The three parameters shown in Table 2 have been used to characterize the fracture mode in engineering materials [8,31,32,33]. In general their sensitivity is attributed to the different proportion between slow transverse (shear) and fast longitudinal waves emitted by the motion of the crack sides under shear or tensile load [36]. In thin structures or materials this could be translated to symmetric and antisymmetric modes, which again exhibit similar changes in velocity [37]. However, other parameters (mainly energy-related) like the “amplitude” and the area under the rectified signal envelope or counts neither yielded similar correlations nor exhibited sensitivity to the mode in this study. From the total number of AE descriptors, RT and RA showed the clearest possible result while no serious correlation was drawn by other descriptors.

**Table 2:** Average values of basic AE parameters for different fracture patterns and sensor locations.

Fracture Type	Specimen Code	AE Channel *	RISE TIME (μs)	A-FRQ (kHz)	RA (μs/V)
Torsion fracture through the body	FRL2	1	82.5	106.9	14544.9
		2	67.7	132.3	6339.3
	FRL11	1	34.5	176.6	6339.4
		2	39.1	91.3	4014.4
	FRL8	1	35.1	129.7	1744.8
		2	31.5	105.0	5342.2
Bending fracture of the head	FRL7	1	3.6	62.9	720.1
		2	10.5	168.3	1503.9
	FRL6	1	18.9	150.8	1659.0
		2	12.6	117.5	1140.2
	FRL1	1	28.4	132.7	2396.6
		2	7.3	226.8	746.3

* The sensor of channel 1 is near the fix point, channel 2 below the femur head.

**Figure 10:**
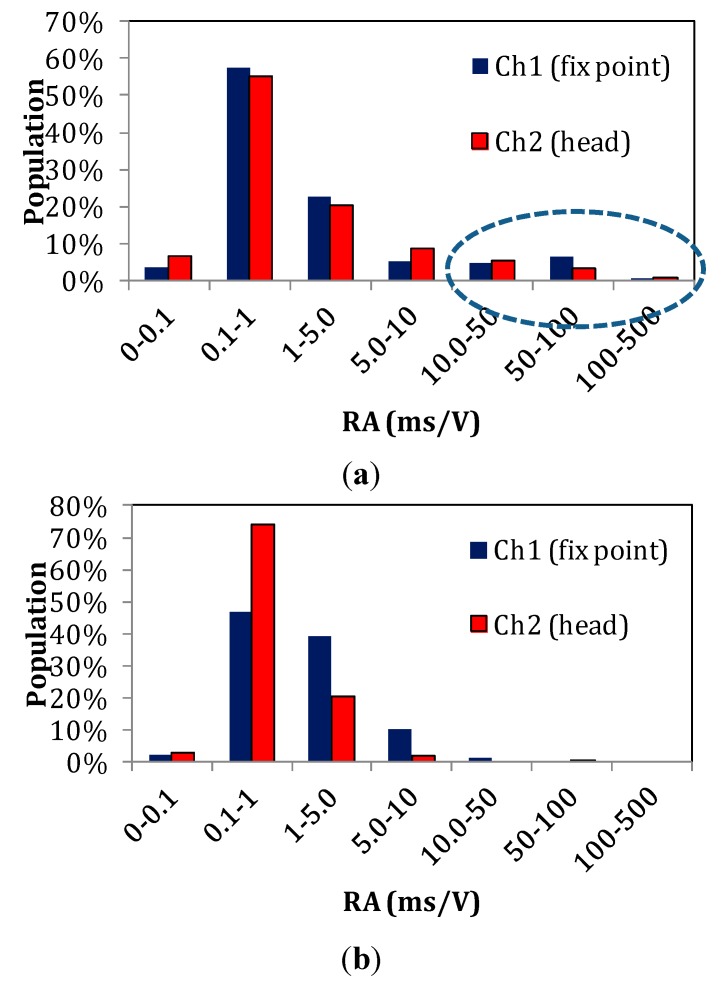
Frequency distribution of RA values for both sensors: (**a**) torsion fracture and (**b**) bending fracture.

Despite the change in average values, there is strong overlap between the populations as seen in Figure 9 above. This is inevitable since fracture is a random phenomenon, passing this randomness on to the corresponding emissions. In many cases populations overlap and the extremes of the populations are used to characterize the processes [38,39] as they modify the population distribution. Figure 10 shows the frequency distribution of the total number of RA values collected at moments of torsion fracture (a) and of bending fracture (b) separately for each channel. In both fracture cases, the values most frequently recorded are between 0.1–1 ms/V. However, for bending fracture, the population of RA values higher than 10 ms/V is nearly zero (1% or less for channels 1 and 2); for torsion fracture, however, the corresponding percentage is approximately 10%—specifically, 11.4% for sensor 1 and 9.4% for sensor 2, as seen in the dashed ellipse in Figure 10a.

The transition to higher RA values has been reported in several cases of engineering materials when fracture is shifting from tensile cracking to shearing. As an example, in bending of concrete beams, the moments of debonding between the external reinforcing patches and concrete are characterized by RA of double value compared to the constant concrete micro-cracking [38]. In addition, fibre pull-out and debonding between fibrous layers of mortar exhibit much higher values of RA compared to early stage cracking due to bending in textile reinforced cement beams [40]. Similar shifts in AE parameters have been obtained in steel bar reinforced concrete specimens, prestressed concrete members, frames and metal plates, under monotonic or cyclic loading, among others [41,42,43,44]. The known behaviour of engineering materials which has been studied more extensively through the years serves as a guideline for interpretation of AE data in bone tissue in relation to the developed loading conditions.

**Figure 11:**
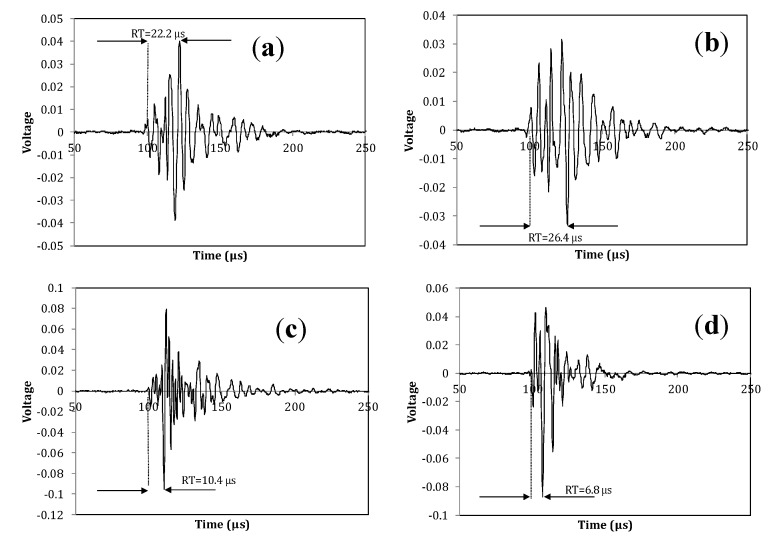
Typical AE waveforms recorded by sensor #2 (beneath the head) during torsion fracture of the diaphysis (**a**,**b**), and bending fracture of the head (**c**,**d**).

Typical full waveforms recorded during moments of fracture can be seen in Figure 11. The first cases (a and b) concern emissions during torsion fracture, while (c and d) involve bending of the neck. The difference in rise time is evident, while the denser cycles imply higher frequency for the bending emissions.

The abovementioned trends str a first step in interpreting the AE signals during fracture in this specific human bone tissue. It seems that the trends learnt from other materials may still be valid for the explanation of such a heterogeneous tissue as human bone. One matter that should always be highlighted in AE studies in such media is the effect of wave propagation. Bone is not a homogeneous and isotropic material system. Therefore, wave propagation interacts with porosity, plate geometry and curvatures. The result is strong dispersion and attenuation which distort the waveform parameters [24,30]. The elastic energy released by the fracture incidents forms wave modes with different speeds and therefore the waveform changes shape as it propagates to the sensor. This means that parameters like RT, RA and AF are changing values throughout propagation. The values recorded by the sensors correspond to the waveforms as received by the sensors at their positions and are not necessarily the same as those emitted by the fracture events. This is the reason that AE values should be compared only between the same sensors so that the propagation conditions, even though not identical, are as similar as possible.

## 4. Conclusions

This paper studies AE activity during fracture of human femur bone tissue. The fracture of this specific bone is common, especially in aged people. Results show that AE activity, as monitored by PZT sensors attached on the surface of the specimens, can be used to determine the start of cracking which occurs much earlier than macroscopic fracture. In the present case, AE activity revealing micro-cracking started at a load as low as one seventh of the maximum load. The increase of rate of recorded signals is a precursor of serious fracture phenomena, while the parameters of the obtained waveforms reveal specific information as to the dominant fracture mechanisms. Specimens which fractured due to torsion exhibited higher percentage of longer AE signals compared to samples that were broken by bending on the femur head. Mechanical and physical properties as well as thickness are also taken into account in an effort to examine the possibility of applying AE methodologies to interpret fracture of bones based on the experience from other engineering materials. Incorporating AE monitoring during mechanical testing of tissue is certainly a challenge with open questions, like sensor positioning and coupling, wave distortion due to microstructure and geometry. However, it can certainly increase the data obtained in areas other than mechanical data alone, and help medical doctors and bioengineers in understanding the fracture of a complicated material such as human bone.
